# B cell depletion or absence does not impede anti-tumor activity of PD-1 inhibitors

**DOI:** 10.1186/s40425-019-0613-1

**Published:** 2019-06-14

**Authors:** William Damsky, Lucia Jilaveanu, Noel Turner, Curtis Perry, Christopher Zito, Mary Tomayko, Jonathan Leventhal, Kevan Herold, Eric Meffre, Marcus Bosenberg, Harriet M. Kluger

**Affiliations:** 1 Departments of Dermatology, Yale School of Medicine, 333 Cedar Street, New Haven, CT 06520 USA; 2 Departments of Medicine, Yale School of Medicine, 333 Cedar Street, New Haven, CT 06520 USA; 3 Departments of Immunbiology, Yale School of Medicine, 333 Cedar Street, New Haven, CT 06520 USA; 4 Departments of Pathology, Yale School of Medicine, 333 Cedar Street, New Haven, CT 06520 USA

**Keywords:** B cell depletion, Immune checkpoint blockade, PD-1, CD20, Melanoma, Colon cancer

## Abstract

**Background:**

PD-1 inhibitors are approved for multiple malignancies and function by stimulating T cells. However, the role of B cells in the anti-tumor activity of these drugs is unknown, as is their activity in patients who have received B cell depleting drugs or with immunoglobulin deficiencies.

**Methods:**

We studied B cell content in 40 melanomas from patients treated with pembrolizumab or nivolumab and assessed the association with response to therapy. Murine MC38 colon cancer and YUMMER1.7 melanoma models were used to determine whether concomitant anti-CD20 antibody injections diminish the anti-tumor effects of anti-PD-1. Results were validated in *mu*MT mice, which lack B cells.

**Results:**

B cells were sparse in most melanomas and B cell content was not associated with response to anti-PD-1 or overall survival. Employing MC38 and YUMMER1.7 models, we demonstrated that anti-CD20 antibodies reduce tumor-infiltrating B cells yet had no effect on tumor growth, response to PD-1 inhibition, or survival. In *mu*MT mice, T-cell dependent tumor rejection and anti-PD-1 responses were no different than in wildtype C57BL/6 J mice.

**Conclusions:**

The degree of tumor infiltrating B cell content is not associated with response to anti-PD-1 inhibitors in melanoma. PD-1 inhibitors cause tumor shrinkage in murine cancer models even when B cells are absent or are depleted. PD-1 inhibitors are likely to be active in patients with impaired B cell function, such as patients undergoing B cell depletion with drugs including rituximab for conditions such as B cell malignancies or autoimmune disorders.

**Electronic supplementary material:**

The online version of this article (10.1186/s40425-019-0613-1) contains supplementary material, which is available to authorized users.

## Introduction

Immune checkpoint inhibitors (CPIs) that block PD-1/PD-L1 or CTLA-4 have transformed the treatment of many cancers and their use continues to expand. Anti-CTLA-4 was approved for treatment of melanoma followed by inhibitors of PD-1 or PD-L1, which have been approved to treat, in addition to melanoma, multiple tumor types [1]. In addition to use as single agents, dual CTLA-4/PD-1 inhibiting regimens have been approved for melanoma and renal cell carcinoma [1].

The anti-tumor mechanisms of action of CPIs involve relief of negative T cell costimulatory signals [2]. However, this effect is not specific to anti-tumor T cells, and the role of B cells, which also express PD-1, in anti-tumor immune response during CPI therapy has not been well established. Activity of CPIs in patients with underlying B cell deficiencies, such as X-linked agammaglobulinemia, immunoglobulin deficiencies, and common variable immunodeficiency has not been studied, as these conditions are rare, and these patients have typically been excluded from clinical trials for oncologic drugs due to their underlying immune disorders.

PD-1 inhibitors have been studied in patients who have been treated with B cell depleting antibodies, such as patients with B cell lymphomas previously treated with rituximab [3]. A number of ongoing clinical trials are assessing the activity of PD-1 inhibitors in combination with rituximab in B cell lymphomas, such as NCT03401853 and NCT02446457, and others. Activity has been seen in clinical trials of PD-1 inhibitors following rituximab therapy [3], yet it remains unknown whether the B cell depletion impedes the anti-tumor activity in this setting. B cell depleting drugs are being used for autoimmune disorders such as rheumatoid arthritis and multiple sclerosis, and the safety of their continued use in patients with malignancies requiring treatment with PD-1 inhibitors has not been explored.

T cell stimulation in many cases is not specific to tumor-infiltrating T cells, and new clinical problems, immune related adverse events (irAEs), have emerged. The precise mechanisms of these irAEs are not well understood, and likely vary by toxicity [4]. PD-1 is expressed on B cells, including immunosuppressive B regulatory cells and has a well-established role in B cell tolerance [5]. Interestingly, changes in B cells induced by checkpoint inhibitors have been shown to specifically correlate with irAEs [6].

High grade (grade 3–4) irAEs are seen in ~ 27% of patients treated with anti-CTLA-4, the most common being hepatotoxicity, dermatitis, and colitis [4]. Grade 3–4 irAEs occur in 10–15% of patients treated with PD-1/PD-L1 inhibitors and 55% of patients treated with the combination [4]. Autoimmune endocrine disorders such as insulin-dependent diabetes, and neurologic disorders such as multiple sclerosis, Guillain-Barre Syndrome and myasthenia gravis can also occur [7–9]. While most irAEs are reversible, some can result in long term morbidities, such as brittle diabetes [9]. Moreover, irAEs can be lethal in the case of pneumonitis and myocarditis; most trials report a treatment related death rate of < 1% for patients treated with anti-PD-1 monotherapy [4]. Although severe irAEs are fairly rare, this highlights the importance of developing appropriate methods to modulate irAEs without impeding anti-tumor effects. Although many autoimmune toxicities are thought to be purely T cell-mediated, some irAEs such as select endocrinopathies, autoimmune hemolytic anemia (AIHA), bullous skin eruptions and some neurologic toxicities appear to be more specifically mediated by B cells through autoantibody secretion. This observation suggests that B cell depletion might be useful a strategy to treat certain irAEs. Limited case reports and/or small series have suggested that anti-B cell therapy can be effective in treating such irAEs (Additional file 1: Table S1).

Given the importance of determining the role of B cells in response to PD-1 inhibitors we first studied B cell density in tumors from melanoma patients treated with PD-1 inhibitors. Subsequently, we employed tumor bearing animal models treated with PD-1 inhibitors alone or with B cell depleting antibodies to assess the role of B cells in anti tumor immune responses. Similar experiments were also performed in *muMT* mice which lack mature B cells. Although we did not study the efficacy of B cell depletion in treating irAEs herein, these data have clear implications for future study of B cell depleting drugs for treating select irAE in patients undergoing CPI therapy.

## Methods

### Patients

Tumor samples from 40 sequential patients with advanced melanoma treated with anti-PD-1 monotherapy (nivolumab or pembrolizumab) with tissue available for study were employed; 34 were evaluable for response. None had pre-existing autoimmune disorders. The median age was 66 years. Twelve patients (30%) were women, 14 (35%) had elevated LDH, four (10%) had mucosal melanoma, and the remainder had cutaneous melanoma. Mutations were found in *KIT* in two patients (5%), in *GNAQ* in one (2.5%), in *NRAS* in nine (22.5%) and in *BRAF* in 12 (30%). Samples were collected pre-treatment on 36 (90%) patients. Areas of invasive melanoma were identified by a board-certified pathologist and cored for generation of a tissue microarray (TMA), incorporating three cores per specimen, using established methods [10]. Specimens and clinical information were retrieved with approval of the Yale University Human Investigations Committee. Patient demographics are summarized in Additional file 1: Table S2. RECIST1.1 was used to classify response. Quantification of B cell content and details of statistical analyses are described in Additional file 1: Supplemental methods.

### Murine studies

YUMMER1.7 and MC38 murine tumor cell lines have been described [11, 12]. Tumor-bearing mice were treated with anti-PD-1 antibody and/or anti-CD20, as described in the Additional file 1: Supplemental methods. Immunohistochemical and flow cytometric analyses are described in the Additional file 1: Supplemental methods.

### Statistics

Associations between B cell content and objective response status was assessed by t-tests. Survival curves were constructed using the Kaplan-Meier method using log-rank statistics. Details are provided in Additional file 1: Supplemental methods.

### Study approval

Studies of human samples were conducted with the approval of a Yale University Institutional Review Board. Written informed consent was obtained from patients. Murine studies were conducted with approval of the Institutional Animal Care and Use Committee.

## Results

**Fig. 1:**
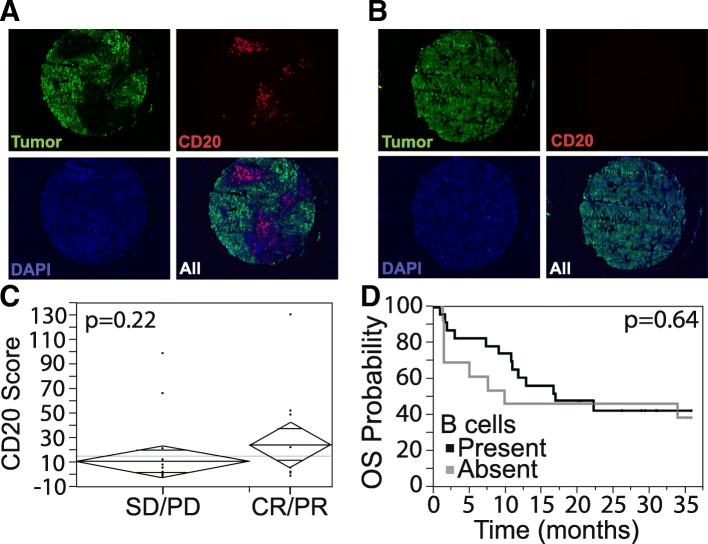
Tumor infiltrating B cell density is not associated with response to anti-PD-1. **a**, **b** Tumor is masked by antibodies to S100 and HMB45 conjugated to Cy2 (green), and the area of B cell infiltrate is determined by the relative area of CD20 positive cells within the tumor mask, identified by anti-CD20 antibodies conjugated to Cy5 (red). Nuclei are identified by DAPI (blue). Examples of high B cell density (**a**) and low B cell density (**b**) are shown. **c** B cell quantification in responders (CR: complete response, PR: partial response) (*n* = 11) vs non-responders (SD: stable disease, PD: progressive disease) (*n* = 23), *p* = 0.22, by unpaired t-test. **d** Presence or absence of tumor infiltrating B cells does not correlate with overall survival, log-rank *p* = 0.64

We studied B cell content in melanoma tumors from 40 patients treated with PD-1 inhibitors. Tumor infiltrating B cells were fairly sparse in most samples, 0–131 cells per 0.6 mm histospot (median - 2.5, mean - 17.4), 3 histospots per patient. Figure 1 A-B shows examples of heavily and poorly infiltrated tumors. Seeing that B cell content can vary within a tumor, we used the highest B cell density in the three histospots to evaluate associations with outcome. Thirty-four patients were evaluable for response using RECIST1.1. The objective response rate was 32%. There was no association between B cell content and radiographic response (Fig. 1C). We defined “high” B cell content as above the median value in at least one histospot and found no difference in overall survival (*P* = 0.64) among tumors with high versus low B cell content by log rank statistics (Fig. 1D).

**Fig. 2:**
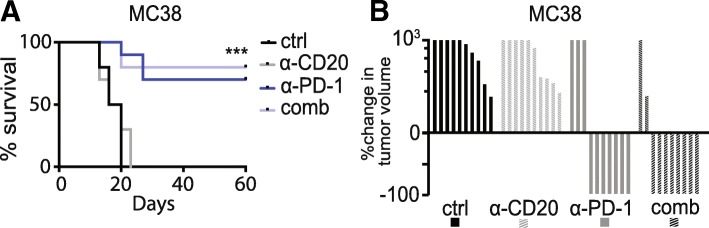
Anti-CD20 therapy does not affect anti-tumor immune responses in murine colon cancer (MC38). **a**. Survival of MC38 tumor-bearing mice treated with saline (ctrl), anti-CD20, anti-PD-1, or anti-CD20 + anti-PD-1 (comb), *n* = 10. Ctrl vs CD20 log-rank *p* = 0.78; PD-1 vs comb *p* = 0.67; ctrl vs comb *p* < 0.0001. **b** Waterfall plot of groups in **a**, shown as percent change in tumor size relative to size at day 10

**Fig. 3:**
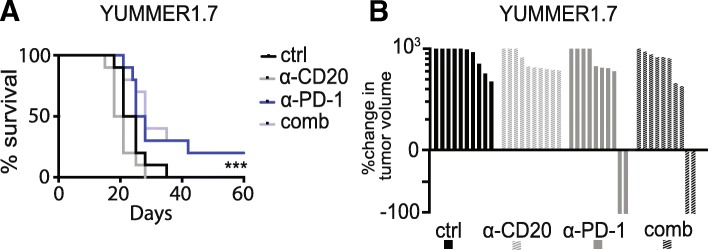
Anti-CD20 therapy does not affect anti-tumor immune responses in murine melanoma (YUMMER1.7). **a** Survival of YUMMER1.7 tumor bearing mice treated with saline (ctrl), anti-CD20, anti-PD-1, or anti-CD20 + anti-PD-1 (comb), *n* = 10. Ctrl vs CD20 *p* = 0.08; PD-1 vs comb *p* = 0.73; ctrl vs comb *p* = 0.0008. **b** Waterfall plot of groups in **a**, shown as percent change in tumor size relative to size at day 10

To determine whether B cell depletion with anti-CD20 antibodies affects anti-tumor efficacy of PD-1 inhibitors, we employed two murine models, MC38 (colon carcinoma) and YUMMER1.7 (melanoma). Tumor-infiltrating B cells were seen at baseline in both models (Additional file 1: Figure S1A-S1D). MC38 tumors are anti-PD-1 sensitive [13]. Anti-PD1 was initiated in mice with established MC38 tumors and followed 2 days later by anti-CD20, which has been shown to effectively deplete murine B cells in blood, spleen, bone marrow and other tissues [14–16]. We had four treatment groups: control, anti-CD20, anti-PD-1, and anti-CD20 + anti-PD-1. Survival of mice (*N* = 10/group) and tumor volume did not differ when anti-CD20 antibodies were added to anti-PD-1 (Fig. 2A-B and Additional file 1: Figure S2A), suggesting that B cells are not required for effective anti-tumor immune responses induced by anti-PD-1 in MC38. Similar results were seen in the YUMMER1.7 (anti-PD-1 sensitive murine melanoma model) using the same experimental design [11]. Anti-CD20 did not affect tumor growth or survival, and did not hamper the anti-tumor effect of anti-PD-1 (Fig. 3A-B, and Additional file 1: Figure S2B). Anti-CD20 antibodies were more effective in reducing B cells in the spleen than in the tumor (Additional file 1: Figure S1E-S1F).

**Fig. 4:**
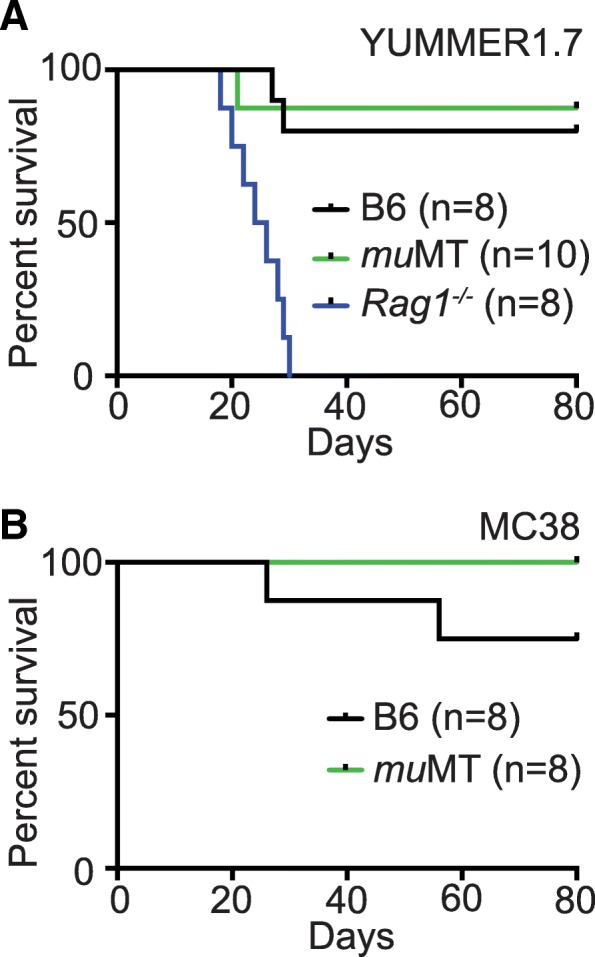
B cell deficiency does not impair anti-tumor immune responses in murine models of colon cancer (MC38) and melanoma (YUMMER1.7). **a** Survival of mice after implantation of 1 × 10^5^ YUMMER1.7 cells. C57BL/6 J (B6) (*n* = 8) vs *mu*MT log-rank *p* = 0.7319 (*n* = 10). *mu*MT vs *Rag1*^*−/−*^ (*n* = 8) *p* < 0.0001. **b** Survival of MC38 tumor bearing mice treated with anti-PD-1. C57BL/6 J vs *mu*MT *p* = 0.1435, *n* = 8

To further test the hypothesis that B cell activity is dispensable for effective anti-tumor responses induced by anti-PD-1, we employed *mu*MT mice that lack B cells due to biallelic inactivation of the *Ighm* gene [17]. We tested the ability of *mu*MT mice to spontaneously reject YUMMER1.7. In this model, tumor rejection is dependent on T cells, as described, but the role of B cells is unknown [11]. When YUMMER1.7 cells were injected into *mu*MT host animals, they rejected YUMMER1.7 cells as efficiently as control mice (Fig. 4A). To test the effect of B cell deficiency in the setting of immunotherapy, we treated both WT and *mu*MT mice bearing MC38 tumors with anti-PD-1. Survival in these groups was similar (Fig. 4B), suggesting that while T cells are required for immune-mediated rejection of tumors, B cells are not.

## Discussion

Our purpose was to determine whether B cells play an essential role in anti-tumor activity of PD-1 inhibitors, and whether use of B cell inhibitors hampers anti-tumor activity of anti-PD-1. While the importance of tumor-infiltrating T cells in anti-tumor immunity is indisputable, the role of B cells is less clear. B cell depletion could affect production of auto-antibodies to melanoma antigens; which could impair anti-tumor immune responses given the observations that in melanoma auto-antibodies have been shown to be associated with improved outcomes, higher tumor-specific CD8 counts and response to ipilimumab [18]. Endogenous immunoglobulins can also regulate antibody dependent cellular cytotoxicity (ADCC) and could be impaired after rixutimab administration. B cells are also known to modulate T cell responses directly by presenting antigens and activating T cells and indirectly by modulating myeloid and/or antigen presenting cell function. Despite all of these considerations, it is unclear if the immune-stimulating role of B cells in advanced melanoma is clinically meaningful, particularly in the setting of metastatic disease and response to anti-PD-1. In humans, CD20 targeting drugs such as rituximab cause apoptosis of mature B cells and lead to an absence of circulating B cells for about 6 months after treatment. However, rituximab spares stem cells and plasma cells and typically does not affect overall immunoglobulin levels [19].

Conversely, some studies have suggested that B cell depletion might actually have anti-cancer effects in certain contexts [20]. For example, anti-CD20 treatment in mice bearing squamous cell carcinoma potentiated the efficacy of chemotherapy and enhanced tumor infiltration by CD8+ T cells [21]. Tumor-associated B cells have even been proposed to promote resistance to BRAF and MEK inhibitors in melanoma through secretion of IGF-1 and clinical trials evaluating therapeutic B cell depletion in this context are underway (NCT01376713) [22]. Immunoregulatory tumor infiltrating B cells (Bregs) have been shown to inhibit anti-tumor T cell responses in some models through IL-10 production [23]. These considerations suggest that B cell depletion might not only be neutral, it might even enrich anti-tumor immunity in certain contexts.

Seeing that core biopsies often do not reflect cellular content of entire tumor, we employed three cores per patient in this study, using the highest value obtained from the cores for our analysis. We found similar B cell content in tumors from responsive and non-responsive patients treated with anti-PD-1, suggesting that elimination of B cells in humans might not significantly affect anti-tumor immunity induced by PD-1 inhibitors. We corroborated this in several murine models. The anti-PD-1-sensitive MC38 model and the UV-mutagenized, neoantigen rich, YUMMER1.7 melanoma model were used. We showed partial B cell depletion with anti-CD20 did not impair anti-tumor immunity in either model. Spontaneous rejection of YUMMER1.7 in *mu*MT mice, which lack B cells, both in tumors and in the circulation and lymphoid organs, was not impaired. Administering anti-PD-1 in *mu*MT mice bearing MC38 or YUMMER1.7 tumors revealed no difference in complete response rate or survival compared to WT C57BL/6 J mice. These data suggest that B cell function may not be strictly required for either effective endogenous or anti-PD-1 elicited anti-tumor immune responses. Further, these results suggest that study of B cell-targeting therapies to treat select B cell-dependent irAE in patients receiving CPI therapy is warranted. It should be noted that despite the absence of mature B cells, *muMT* mice have been shown in some studies to still produce some immunoglobulins [24].

As immune checkpoint inhibitors are becoming widely used in multiple tumor types, they are also being used in B cell malignancies. Many patients with B cell malignancies have received B cell eliminating drugs, raising the question of whether this is determental for anti-tumor activity. Moreover, use of PD-1 inhibitors in patients with underlying autoimmune disorders treated with B cell eliminating drugs who develop cancer has not been studied, although case-series suggest that in some it is tolerated [25]. Once CPIs are initiated in patients with underlying autoimmunity, their disease might flare. Our data from the MC38 and YUMMER 1.7 models suggest that use of rituximab in this setting might not be detrimental and requires further study in humans. Finally, our results from the *mu*MT mice bearing MC38 or YUMMER1.7 tumors suggests that patients with underlying B cell deficiencies or prior therapy with B cell depleting drugs might similarly benefit from PD-1 inhibitors shold they develop malignancies.

With widespread use of CPIs for malignancies, the incidence of toxicities will likely rise, and previously undescribed irAEs, such as cardiac toxicities, have only recently been reported [26]. Although it is unclear whether mechanisms of classic autoimmune disorders are exactly recapitulated in patients treated by CPIs who develop autoimmunity, diseases such as AIHA and bullous pemphigoid which are bonafide antibody-mediated disorders, have been shown to to respond to rituximab when triggered by CPI [27, 28]. Others, such as autoimmune diabetes, rheumatoid arthritis, type I diabetes mellitus, and multiple sclerosis are thought to be predominantly T cell mediated. However, anti-B cell therapy in classic rheumatoid arthritis and multiple sclerosis have shown efficacy, further supporting the study of anti-CD20 antibodies in treating these irAE. Several groups have already reported successful use of rituximab for treatment of B-cell mediated irAEs as summarized in Additional file 1: Table S1.

In summary, this is the first study look for a potential association between B cell infiltrates and benefit from PD-1 inhibitors in melanoma. Although B cell content appeared to be slightly higher in patients who responded to anti-PD-1 than patients who did not, this did not reach statistical significance in this cohort. Furthermore, using two, well-established murine cancer models we found that B cell inhibition did not affect T cell dependent anti-tumor responses induced by PD-1 inhibitors in these models. Taken together, these studies support further research into the use and impact of anti-B cell therapy in patients receiving CPIs who have underlying B cell deficiencies, have received B cell depleting antibodies for underlying autoimmune disorders or B cell malignancies, or patients with select irAEs induced by PD-1 inhibitors.

## Additional file

Additional file 1: Supplemental methods, Table S1-S2 and Figure S1-S2. (DOCX 1075 kb)
