# Supplementary material for: B cell depletion or absence does not impede anti-tumor activity of PD-1 inhibitors
Source: J Immunother Cancer. 2019 Jun 14;7:153. doi: 10.1186/s40425-019-0613-1 (PMC6567557; doi:10.1186/s40425-019-0613-1)
Supplement: Supplementary file 1 — Supplemental methods, Table S1-S2 and Figure S1-S2. (DOCX 1075 kb) [file 40425_2019_613_MOESM1_ESM.docx]

B cell depletion or absence does not impede anti-tumor activity of PD-1 inhibitors

Additional file 1: Supplemental methods, Table S1-S2 and Figure S1-S2

**Supplemental Methods**

Quantification of B cell content in human tumors: Immunofluorescence staining was performed as described.(1, 2) Staining for CD20 was performed with a mouse monoclonal antibody (clone L26, cat#M0755, Dako). Tumor area was identified with a rabbit anti-S100 antibody (cat# Z0311, Dako). CD20 positive cells were counted within the tumor mask in each histospot. Tumor spots were included if they contained sufficient tumor (>3% of the histospot area) and were not necrotic.

Murine tumor models

Tumor cells are were implanted subcutaneously into the right hind flank of wildtype C57BL/6J, *mu*MT (*Ighm^-/-^)*, or *Rag1^-/-^* (Jackson Laboratory) host animals. For the YUMMER1.7 spontaneous tumor rejection model 1x10^5^ cells were implanted, whereas for the tumor formation model, 5x10^5^ cells were implanted as previously described.(3) For MC38, 2.5x10^5^ cells were implanted. Mice were euthanized according to humane endpoints when tumors reached 1.5cm in maximum dimension, or in rare cases sooner for tumor ulceration.

Murine drug treatments: Anti-mouse PD-1 antibody (RMP1-14) (Bio X Cell) therapy was initiated on day 7 after tumor implantation when tumors were roughly 50-100mm^3^. Anti-PD-1 was diluted in sterile saline and administered by intraperitoneal injection at 8 mg/kg every 3 days for a total of 6 doses. Anti-mouse CD20 antibody (AISB12) (Bio X Cell) therapy was initiated on day 9 (3 days after anti-PD-1 therapy) and administered at 25 mg/kg in sterile saline by intraperitoneal injection on two consecutive days. Anti-CD20 therapy was similarly repeated every 2 weeks. Saline vehicle was administered to control animals.

Immunohistochemistry: Murine tumors were harvested at experimental endpoint, formalin fixed, and paraffin-embedded. Immunohistochemistry was performed using standard methods and anti-mouse B220 antibody. Intratumoral B cell density (number of B220 positive cells/high power field HPF) was assessed when tumors were ~500mg (0.75cm in largest dimension) for 10 HPF/tumor and the mean was calculated using 3 replicate animals/group.

Tumor dissociation and flow cytometry: Tumors for flow cytometry were minced in RPMI with 2% FBS, 0.5mg/mL collagenase IV and 200mg/L DNase, digested in a 37C incubator for 30 minutes, and filtered to remove debris. Single-cell suspensions from tumors or splenocytes were incubated with anti-Fc receptor antibody (2.4G2) on ice for 15 minutes. Cells were stained with appropriate antibodies in 2.4G2-containing buffer. Data was acquired on an LSR II (BD Biosciences) and analyzed with FlowJo. LIVE/DEAD discrimination was performed with LIVE/DEAD red from Invitrogen. Antibodies against CD45 (A20) were obtained from eBioscience, antibodies against CD3 (17A2), CD11b (M1/70), and CD20 (SA275A11) were obtained from Biolegend, and antibodies against B220 (RA3-6B2) were obtained from BD Biosciences.

Statistical analyses: JMP version 5.0 software was used. The association between B cell content and objective response status (complete response (CR) and partial response (PR) versus stable disease (SD) and progressive disease (PD) was assessed by t-tests. Survival curves were constructed using the Kaplan-Meier method. The log-rank test was used to test the significance in distribution between patients with high or low B cell content. For murine data, Prism version 7.0 software was used. Difference in survival was assessed using the Kaplan-Meier method and statistical significance was determined using Log-rank (Mantel-Cox).

**Table S1. Reported cases of rituximab use to treat irAE induced by immune checkpoint inhibitors (ICI).** *ITP; immune thrombocytopenic purpura.

| **Author** | **Tumor type** | **ICI** | **irAE** | **Other immune suppression** | **irAE response** | **Tumor response** |
| --- | --- | --- | --- | --- | --- | --- |
| Williams(4) | Melanoma | Nivolumab + ipilimumab | Encephalitis | Steroids + IVIg | Yes | PR |
| Ito(5) | Small cell lung cancer | Nivolumab + ipilimumab | Encephalitis | Steroids + IVIg | Yes | PD |
| Khan(6) | Melanoma | Nivolumab + ipilimumab | Autoimmune hemolytic anemia | Steroids | Yes | SD |
| Sowerby(7) | Non-small cell lung cancer | Nivolumab | Bullous pemphigoid | Steroids | Yes | CR |
| Padda(8) | Melanoma | Ipilimumab | Vasculitis | Steroids | Yes | Not reported |
| Shiuan(9) | Melanoma | Ipilimumab | ITP* | Steroids + IVIg + romiplostim | Yes | Not reported |
| Shiuan(9) | Melanoma | Ipilimumab | ITP | Steroids + IVIg | Yes | Not reported |
| Shiuan(9) | Melanoma | Nivolumab + ipilimumab | ITP | Steroids + IVIg | Not reported | PR |
| Hasanov (10) | Urothelial  carcinoma | Nivolumab | Cold agglutinin syndrome | Steroids | Yes | Not reported |
| Shaikh  (11) | Lung adenocarcinoma | Nivolumab | Autoimmune hemolytic anemia | Steroids | Yes | SD |
| Ridpath  (12) | Melanoma | Nivolumab | Bullous pemphigoid | Steroids  Plasma exhange | Yes | Not reported |
| Ghosn  (13) | Melanoma | Pembrolizumab | Neuro-Sjogren’s syndrome | Steroids  IVIG | Yes | CR |
| Crusz  (14) | Melanoma | Pembrolizumab | Myasthenia  Gravis | Steroids  Mycophenolate  Plasma exhange | Yes | Not reported |

**Table S2. Baseline Characteristics of anti-PD1 Treated Patients with Melanoma (N=40)**

| **Characteristic** | **n** | **%** |
| --- | --- | --- |
| Age, years |  | |
| Median | 66 | |
| Range | 43-92 | |
| Sex |  | |
| Male | 28 | 70 |
| Female | 12 | 30 |
| Elevated LDH | 14 | 35 |
| Mutation |  | |
| *BRAF* | 12 | 30 |
| *NRAS* | 10 | 25 |
| *c-Kit* | 2 | 5 |
| *GNAQ* | 1 | 0.25 |
| Primary melanoma type |  | |
| Cutaneous non-acral lentiginous | 31 | 77.5 |
| Acral lentiginous | 4 | 10 |
| Mucosal | 4 | 10 |
| Uveal | 1 | 2.5 |

**
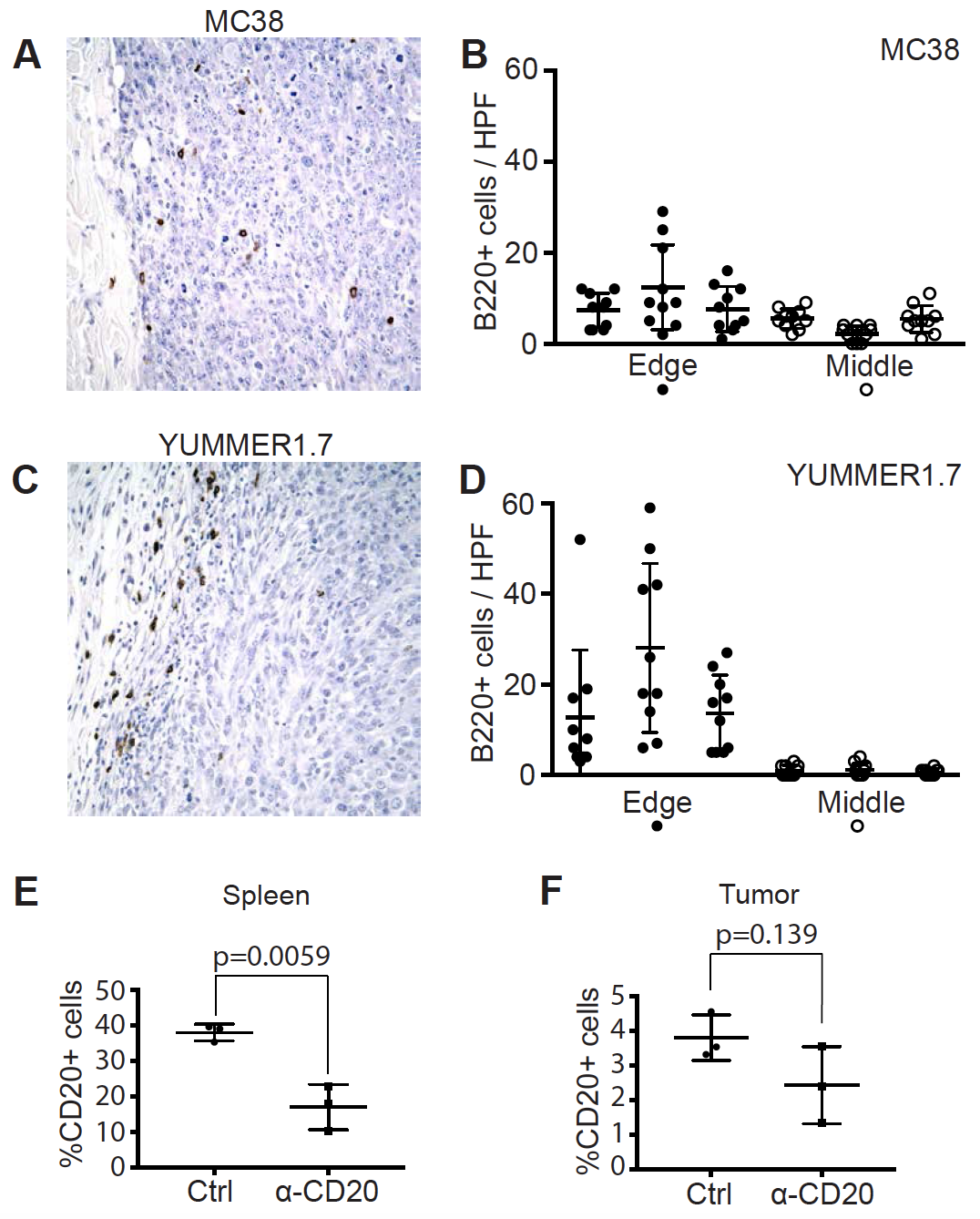
**

**Figure S1.** A. Tumor infiltrating B cell density in MC38. B. Quantification of A. C. Tumor infiltrating B cell density in YUMMER1.7. D. Quantification of C. E. Quantification of flow cytometry experiments showing number of CD20+ cells in spleen (shown as percentage of all lymphocytes). F. Quantification of flow cytometry experiments showing number of CD20+ cells in tumor (shown as percentage of all lymphocytes). For E and F, note that a different anti-CD20 antibody clone was used to measure depletion (SA275A11) than was used for depletion (AISB12).

**
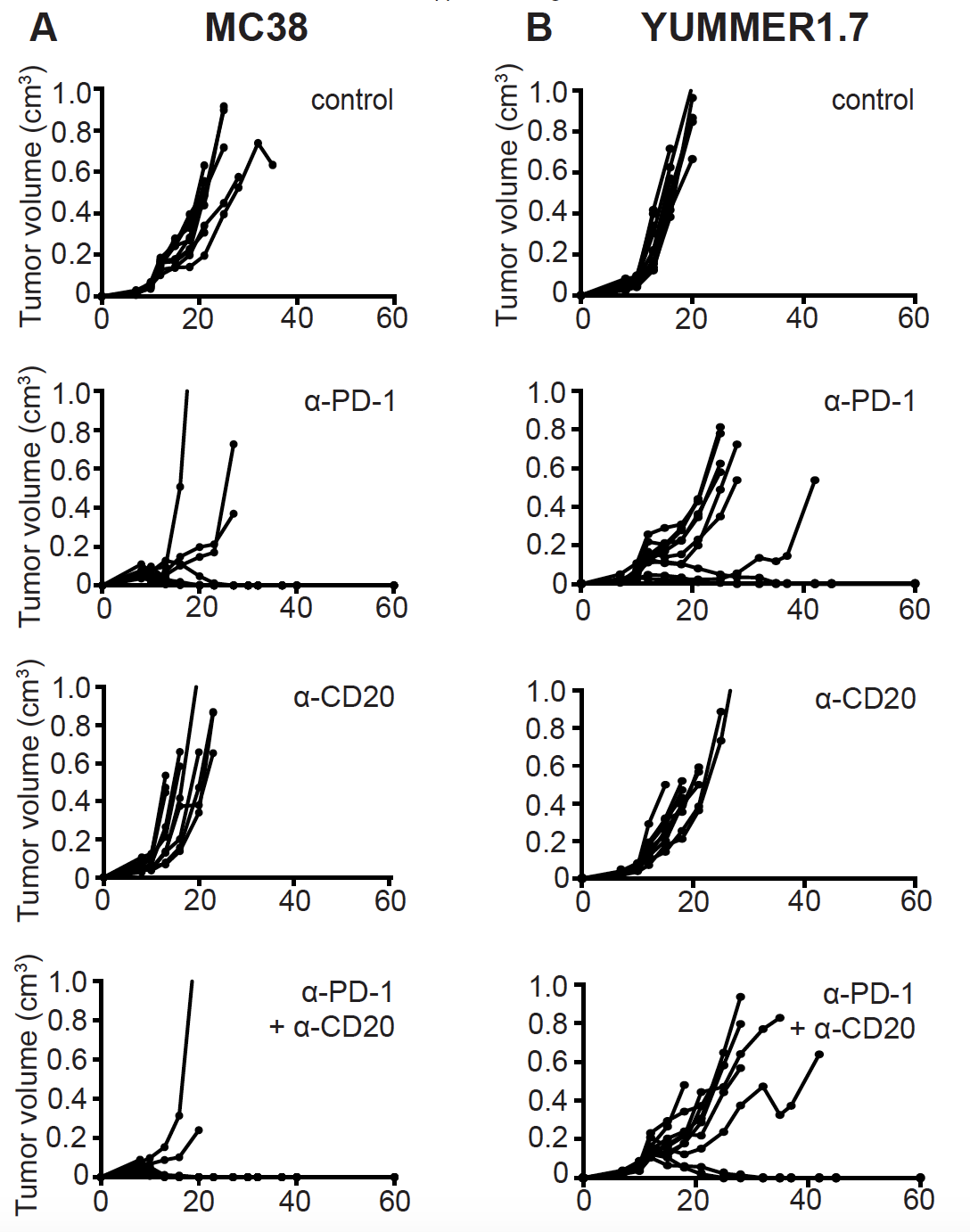
**

**Figure S2.** A. Spider plots showing MC38 tumor growth in individual mice from Figures 3A and 3B. B. Spider plots showing YUMMER1.7 tumor growth in individual mice from Figures 3C and 3D.

**Supplemental References**

1. Baine MK, Turcu G, Zito CR, Adeniran AJ, Camp RL, Chen L, et al. Characterization of tumor infiltrating lymphocytes in paired primary and metastatic renal cell carcinoma specimens. Oncotarget. 2015;6(28):24990-5002.

2. Kluger HM, Zito CR, Barr ML, Baine MK, Chiang VL, Sznol M, et al. Characterization of PD-L1 Expression and Associated T-cell Infiltrates in Metastatic Melanoma Samples from Variable Anatomic Sites. Clin Cancer Res. 2015;21(13):3052-60.

3. Wang J, Perry CJ, Meeth K, Thakral D, Damsky W, Micevic G, et al. UV-induced somatic mutations elicit a functional T cell response in the YUMMER1.7 mouse melanoma model. Pigment Cell Melanoma Res. 2017;30(4):428-35.

4. Williams TJ, Benavides DR, Patrice KA, Dalmau JO, de Avila AL, Le DT, et al. Association of Autoimmune Encephalitis With Combined Immune Checkpoint Inhibitor Treatment for Metastatic Cancer. JAMA Neurol. 2016;73(8):928-33.

5. Ito M, Fujiwara S, Fujimoto D, Mori R, Yoshimura H, Hata A, et al. Rituximab for nivolumab plus ipilimumab-induced encephalitis in a small-cell lung cancer patient. Ann Oncol. 2017;28(9):2318-9.

6. Khan U, Ali F, Khurram MS, Zaka A, Hadid T. Immunotherapy-associated autoimmune hemolytic anemia. J Immunother Cancer. 2017;5:15.

7. Sowerby L, Dewan AK, Granter S, Gandhi L, LeBoeuf NR. Rituximab Treatment of Nivolumab-Induced Bullous Pemphigoid. JAMA Dermatol. 2017;153(6):603-5.

8. Padda A, Schiopu E, Sovich J, Ma V, Alva A, Fecher L. Ipilimumab induced digital vasculitis. J Immunother Cancer. 2018;6(1):12.

9. Shiuan E, Beckermann KE, Ozgun A, Kelly C, McKean M, McQuade J, et al. Thrombocytopenia in patients with melanoma receiving immune checkpoint inhibitor therapy. J Immunother Cancer. 2017;5:8.

10. Hasanov M, Konoplev SN, Hernandez CMR. Nivolumab-induced cold agglutinin syndrome successfully treated with rituximab. Blood Adv. 2018;2(15):1865-8.

11. Shaikh H, Daboul N, Albrethsen M, Fazal S. A case of autoimmune haemolytic anaemia after 39 cycles of nivolumab. BMJ Case Rep. 2018;2018.

12. Ridpath AV, Rzepka PV, Shearer SM, Scrape SR, Olencki TE, Kaffenberger BH. Novel use of combination therapeutic plasma exchange and rituximab in the treatment of nivolumab-induced bullous pemphigoid. Int J Dermatol. 2018;57(11):1372-4.

13. Ghosn J, Vicino A, Michielin O, Coukos G, Kuntzer T, Obeid M. A severe case of neuro-Sjogren's syndrome induced by pembrolizumab. J Immunother Cancer. 2018;6(1):110.

14. Crusz SM, Radunovic A, Shepherd S, Shah S, Newey V, Phillips M, et al. Rituximab in the treatment of pembrolizumab-induced myasthenia gravis. Eur J Cancer. 2018;102:49-51.
